# Emerging microbiome-directed therapies in inflammatory bowel disease: beyond diet modification and FMT

**DOI:** 10.1007/s00281-025-01066-5

**Published:** 2025-11-25

**Authors:** Andrea Carolina Quiroga-Centeno, Konstantina Atanasova, Matthias Philip Ebert, Anne Kerstin Thomann, Wolfgang Reindl

**Affiliations:** 1 Department of Medicine II, Medical Faculty Mannheim, Heidelberg University, Mannheim, Germany; 2 Department of Surgery, Universidad Industrial de Santander, Bucaramanga, Colombia; 3 DKFZ-Hector Cancer Institute at the University Medical Center, Mannheim, Germany; 4 Molecular Medicine Partnership Unit, European Molecular Biology Laboratory, Heidelberg, Germany

**Keywords:** Gastrointestinal microbiome, Dysbiosis, Inflammatory bowel disease, Therapy, Precision medicine

## Abstract

Inflammatory bowel disease (IBD) is a multifactorial and heterogeneous disorder that remains challenging to manage. Growing evidence implicates the gut microbiome as a key player in IBD pathogenesis, with many patients displaying intestinal dysbiosis that can drive aberrant immune responses. Traditional microbiome-targeted interventions, such as dietary modifications, probiotics, and fecal microbiota transplantation (FMT), have yielded mixed and often temporary benefits in IBD. This shortcoming of broad-spectrum approaches underscores the need for more precise, personalized strategies that account for each patient’s unique microbiota and disease phenotype. Recent advances in omics and bioengineering have catalyzed the development of emerging microbiome-directed therapies that move beyond these broad approaches. This narrative review highlights emerging microbiome-directed therapies that aim to restore gut homeostasis and mitigate inflammation in IBD. We critically evaluate the rationale and therapeutic potential of rationally designed bacterial consortia and genetically engineered bacteria, which represent next-generation probiotics tailored to complement deficient microbial functions or deliver anti-inflammatory agents in situ. We also expand the discussion to underexplored microbiome constituents – archaea, protists, bacteriophages, and fungi – highlighting their roles in IBD and potential as therapeutic targets. Finally, we discuss the key advances and ongoing challenges of these innovative approaches, from ecological stability and engraftment to safety and regulatory considerations.

## Introduction

IBD encompasses chronic inflammatory conditions of the gastrointestinal tract, primarily ulcerative colitis (UC) and Crohn’s disease (CD), arising from an inappropriate mucosal immune response in genetically susceptible individuals [1, 2]. Globally, IBD incidence and prevalence have risen steadily, imposing significant healthcare burdens [3]. Despite the availability of numerous anti-inflammatory and immunosuppressive therapies (including corticosteroids, biologics, and small molecules), a significant proportion of patients do not achieve sustained remission [4]. This therapeutic gap has prompted intense interest in alternative strategies that target the underlying drivers of inflammation beyond the immune system.

One such driver is the gut microbiota, which encompasses bacteria, viruses (including bacteriophages), fungi, protists, and archaea, playing a crucial role in immune education, nutrient metabolism, and mucosal barrier maintenance [5]. Dysbiosis has been consistently linked with IBD pathogenesis [5–7]. IBD-associated dysbiosis is typically characterized by reduced overall microbial diversity, depletion of beneficial commensals (e.g. butyrate-producing *Faecalibacterium prausnitzii*), and expansion of potential pathogens (“pathobionts”). For instance, adherent-invasive *Escherichia coli* and *Klebsiella pneumoniae* are enriched in some IBD patients and can drive intestinal inflammation in experimental models [8–10]. These microbial shifts correlate with mucosal immune dysregulation and disease activity. For example, diminished levels of microbiota-derived short-chain fatty acids are associated with epithelial damage, whereas increases in regulatory T-cells and anti-inflammatory cytokines (e.g. IL-10, TGF-β) accompany therapeutic improvement [11, 12]. While causality remains under investigation, studies in germ-free mice have demonstrated that colonization with microbiota from IBD patients can increase host susceptibility and, in recent evidence, is sufficient to elicit colitis-like mucosal inflammation, reinforcing a mechanistic link between dysbiosis and disease pathogenesis [13, 14].

Given this pathogenic interplay, restoring the microbiota has become a therapeutic goal. Early interventions to “reset” the microbiome – including diet changes and supplementation with probiotics, prebiotics, or synbiotics – have shown limited success in IBD, often yielding only transient clinical improvements [12]. Though these interventions have had limited and inconsistent success in achieving clinical remission, they have provided valuable insights into host–microbe interactions and paved the way for more innovative treatments. Notably, fecal microbiota transplantation (FMT) has demonstrated that modifying the gut microbiota can induce remission in UC: in randomized trials, multi-donor FMT induced clinical remission in roughly 24–32% of UC patients at 8 weeks, compared to ~ 5–10% on placebo [15–19]. Additionally, higher engraftment of donor strains has been associated with the maintenance of prolonged remission, underlining the microbiome’s functional impact [15–17, 20]. However, FMT outcomes are highly variable and often donor-dependent, with the intriguing “super-donor” phenomenon where only stool from certain donors achieves efficacy [21, 22]. The optimal donor characteristics remain unclear, and recent analyses indicate that complex donor–recipient microbial interactions (including specific strain-to-strain crosstalk) influence FMT success [23–25]. Moreover, practical limitations of FMT, such as the need for repeated administrations, regulatory concerns, and risk of transmitting pathogens, also limit its broad applicability [26]. These shortcomings underscore the persistent gap in our therapeutic arsenal and the need for more targeted and personalized microbiome therapies. Thanks to advances in multi-omics and microbial culture techniques, it is now possible to characterize the gut microbiome in unprecedented detail and design interventions at a finer scale. In this review, we focus on these emerging microbiome-directed therapies that move beyond broad interventions.

## Methodology

### Literature search strategy

We conducted a comprehensive literature search to identify studies on emerging microbiome therapies in IBD. Searches were performed in PubMed and Embase for articles published up to July 2025. Key search terms included combinations of “microbiome”, “inflammatory bowel disease”, “IBD”, “gut microbiota”, “fecal transplant”, “bacterial consortium”, “probiotic engineering”, “bacteriophage therapy”, “archaea IBD”, “mycobiome”, and “protists IBD”. We included primary research articles (preclinical and clinical studies) as well as high-quality review papers and meta-analyses that provided mechanistic insights or clinical data on microbiome-directed interventions. Reference lists of relevant papers were hand-searched to identify additional studies. Non-English language papers were excluded unless a translation was available. Given the narrative nature of this review, our goal was to capture the range of emerging therapeutic strategies rather than providing an exhaustive catalogue of every publication.

## Rationally designed microbial consortia

Given the limitations of crude fecal transplants, researchers have pursued a more controlled approach by developing rationally designed microbial consortia – mixtures of specific bacterial strains formulated to repair dysbiosis and induce anti-inflammatory effects. This strategy draws inspiration from FMT successes but aims to eliminate the unpredictability of stool donors by using a standardized, lab-cultured set of bacteria. These consortia, delivered as oral live biotherapeutic products (LBPs), are composed of commensal bacteria selected for defined functional traits and ability to engraft in the host gut [27]. The underlying principle is to replenish microbes that are deficient in IBD and provide functions that promote mucosal homeostasis (e.g. SCFA production, reinforcement of the epithelial barrier, immune regulation) [28]. A proof-of-concept for LBP efficacy comes from recurrent *C. difficile* infection: a consortium product (RBX2660) received FDA approval after demonstrating superiority over placebo in preventing *C. difficile* recurrence [29]. The IBD consortia under development are based on the observations that certain communities of gut bacteria, particularly clusters of *Clostridia* with immunoregulatory properties, can protect against colitis in preclinical models [11, 30]. Notably, a 2013 landmark study by *Atarashi* et al. showed that colonizing mice with a mix of 17 *Clostridium* strains (isolated for their ability to induce regulatory T cells) ameliorated colitis severity [11]. This finding suggested that defined bacterial cocktails could confer similar benefits as FMT but in a more reproducible manner.

### From enrichment to rational design

Early efforts like the *Atarashi* consortium used a top-down enrichment approach: starting from a fecal sample and enriching for beneficial strains (e.g., spore-forming *Clostridia*) that drive IL-10 ^+^ Tregs [11]. While effective in concept, such enrichments can yield different results depending on the donor sample and may inadvertently include uncharacterized or undesirable strains. Newer consortia development has shifted toward a bottom-up design, selecting strains from scratch based on genomic and metabolic knowledge to cover key therapeutic functions. *Van der Lelie* et al. (2021) pioneered this approach by computationally modeling microbial interactions and nutrient networks to assemble consortia named GUT-103 (17 strains) and GUT-108 (11 strains) [28]. These consortia include not only Clostridial species (clusters IV, XIVa, XVIII) but also other genera like *Bacteroides* and *Akkermansia* to provide functions missing from *Clostridia* alone (such as production of propionate, tryptophan metabolites, and bile-acid transformations). Moreover, the designers paid attention to strain-level selection: genomes were screened to pick strains with complementary nutrient requirements (auxotrophies) so they would cooperate rather than compete within the consortium, which would prevent one strain from overgrowing and ensure stability. Furthermore, multi-genome comparisons in the GUT-103/108 project revealed that species historically labeled as single entities (e.g. *Faecalibacterium prausnitzii*) actually comprise multiple genomically distinct species [28]. Thus, rational consortium design must operate at the strain (or even subspecies) level to ensure the inclusion of the right microbial players. Higher-order community modeling, incorporating beyond-pairwise interactions, is also being explored to predict which strain combinations will remain stable and functional in vivo [31]. This approach, informed by ecology and systems biology, represents a sophisticated evolution from earlier empiric mixtures.

### Preclinical evidence

Rationally designed consortia have shown encouraging preclinical efficacy in IBD models. The 17-strain *Clostridia* mix from 2013 not only increased colonic Treg levels but also significantly reduced colitis severity in mice [11]. *Van der Lelie*’s GUT-103 and GUT-108 consortia demonstrated robust disease mitigation in gnotobiotic mouse models of chronic immune-mediated colitis. Specifically, GUT-103 colonization restored disrupted microbial metabolic functions and prevented colitis onset in susceptible mice, while GUT-108 was able to treat established colitis, achieving reductions in inflammatory markers and histologic damage comparable to healthy controls [28]. Notably, these consortia also drove down levels of opportunistic pathogens (e.g. *Enterobacteriaceae*) and increased beneficial taxa, reflecting a broad correction of dysbiosis. Mechanistically, treated mice exhibited normalization of luminal SCFA concentrations and bile acid profiles, and immunologically, an increase in IL-10-producing Tregs alongside reduced pro-inflammatory cytokines was observed (paralleling the protective immune shifts seen in the earlier *Clostridia* studies). These preclinical successes have provided a rationale to advance consortia to human testing.

### Clinical development

Several defined consortia are now in clinical trials for IBD. One example is VE202, an LBP composed of multiple *Clostridia* strains, which completed Phase 1 testing and has entered a Phase 2 trial in patients with mild-to-moderate UC. Although detailed results are pending publication, Phase 1 data indicated a good safety profile and evidence of engraftment in participants’ microbiomes [32, 33]. Given its potent activity in animal studies, GUT-108 consortium (now under development by a biotechnology company) is also expected to progress to human trials. Importantly, an obstacle in clinical translation is ensuring that lab-grown strains can robustly engraft in the inflamed gut environment of IBD patients, which may be hostile due to factors like altered pH, nutrients, and concurrent medications. The consortium design process explicitly addressed this by including hardy spore-formers and resource-sharing networks among strains [33, 34]. Early human studies have shown that multi-strain probiotics or consortia typically colonize transiently unless given continuously, but there have been instances of long-term persistence of specific strains from consortia, especially if they fill an ecological niche in the host. An interesting finding from a phase 1b trial of a 9-strain consortium (SER-287) in UC was that higher doses led to greater microbiome engraftment and a trend toward clinical remission, although a subsequent phase IIb study did not meet its primary endpoint [34, 35]. Some consortia developers are also exploring synbiotic formulations (combining the consortium with specific prebiotic fibers) to enhance engraftment by providing the introduced strains with a competitive advantage in the gut [36].

### Clinical outlook

#### Current standing and therapeutic potential

Rational microbial consortia are a promising middle-ground between single-strain probiotics and the complexity of FMT. By using a defined composition, they offer significant advantages in consistency, dosing control, and the ability to meet pharmaceutical manufacturing standards. A key benefit is enhanced safety and quality control; unlike FMT, which carries a risk of transmitting pathogens or undesirable genes, defined consortia use well-characterized strains that can be screened to remove pathogenic factors. For instance, identifying a transferable vancomycin-resistance element in a candidate strain (*Blautia coccoides*) highlights the imperative of whole-genome sequencing, which allows manufacturers to exclude or modify such strains to mitigate risks [37]. On the regulatory side, these products are evaluated as live biotherapeutics, similar to drugs, and the FDA’s 2023 guidance on microbiome therapies indicates a clear pathway forward. The prior approval of the RBX2660 consortium for *C. difficile* sets a valuable precedent that could ease the path for IBD-focused consortia.

#### Key challenges and hurdles

Despite the clear potential, key challenges remain. One major hurdle is efficacy optimization, which requires a deeper understanding of which specific microbial functions correlate with clinical remission in human IBD. Another significant challenge is achieving durable engraftment in the host gut, with some patients potentially requiring maintenance dosing to ensure the consortium’s persistence and functional activity.

#### Future directions and vision

**Fig. 1:**
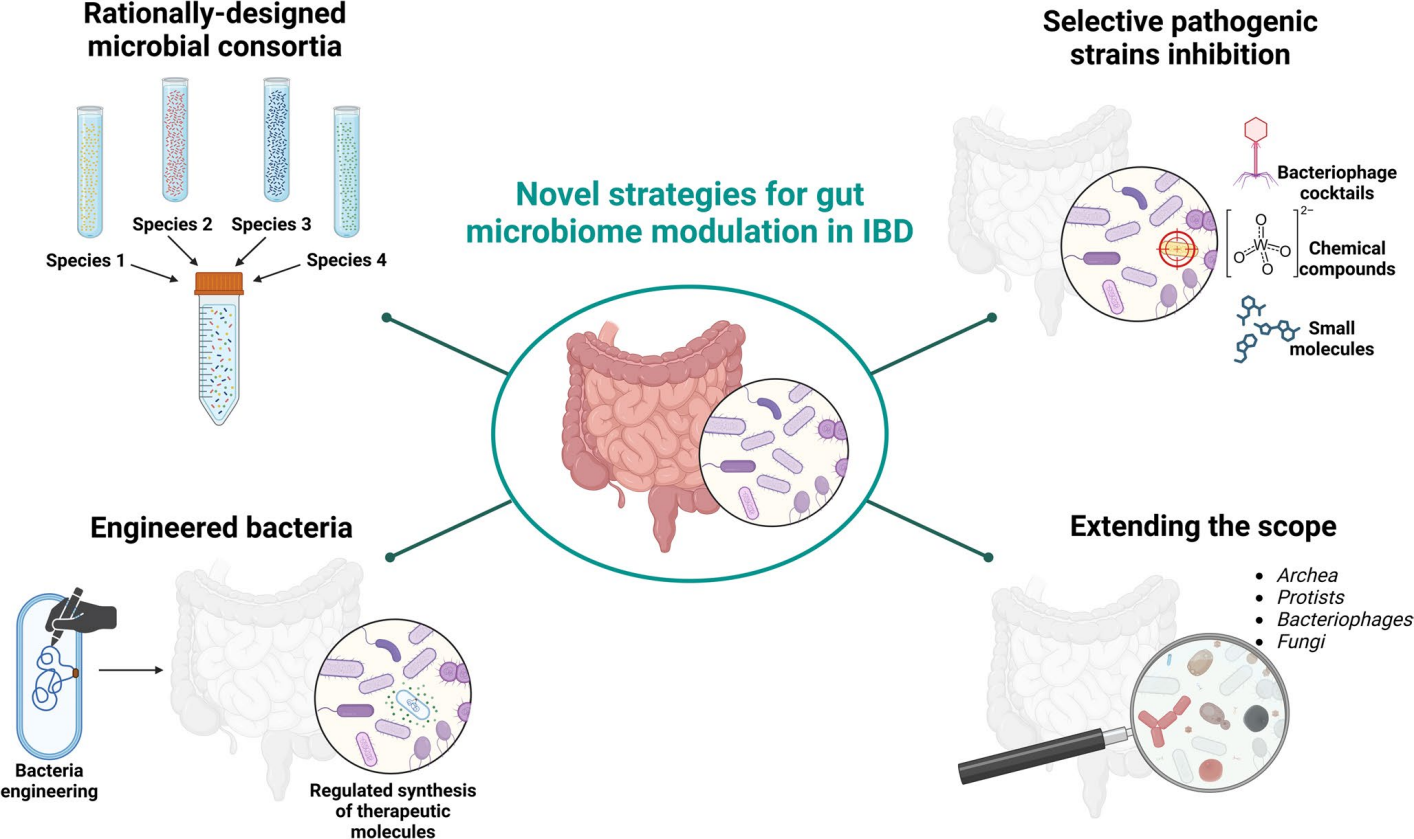
Comparative overview of emerging microbiome-directed therapies, highlighting the differences in their approach towards microbiome modulation. Rational consortia (left) introduce beneficial microbes to restore diversity and function, whereas targeted inhibition and phage therapies (center) remove or suppress specific pathogens. Engineered bacteria (right) act as “living drugs,” delivering anti-inflammatory factors. While consortia broadly reshape community composition, pathogen-targeted and engineered microbes have narrower, more specific actions. Created with BioRender

Looking forward, the application of consortia is expected to be highly adaptable and integrated into broader treatment regimens. If efficacy is proven, consortia could be tailored to different IBD phenotypes, such as using a consortium rich in butyrate-producers for patients with barrier dysfunction [28]. This adaptability extends to regional modifications or even true personalization based on an individual’s microbiome deficits, though this remains a longer-term vision. It is possible that some consortia will require the addition of personalized components for certain patients to achieve optimal outcomes. Ultimately, consortia might be most effective not as a monotherapy but in combination with existing IBD therapies. For example, they could be used alongside biologics to help maintain drug-free remission once inflammation is controlled, representing a multi-faceted approach to restoring gut health.In the following sections, we’ll contrast this additive approach with strategies that subtract or modify specific microbiome components (Fig. 1).

## Selective pathogenic strain Inhibition

While the restoration of the microbiota as a whole is one approach, an alternative strategy is to selectively eliminate or neutralize pathogenic members of the community that drive inflammation. The goal is to precisely suppress harmful bacteria (or their virulence mechanisms) in IBD, without causing collateral damage to beneficial microbes as conventional antibiotics do. This concept stems from observations that certain bacteria (e.g., adherent-invasive *E. coli* (AIEC) in ileal Crohn’s disease, or sulfate-reducing bacteria in UC) disproportionately contribute to mucosal injury and immune activation. Targeting these culprits could rebalance the microbiota and reduce inflammation. Three main modalities are under exploration: metabolic pathway inhibitors, adhesin/receptor blockers, and bacteriophage therapy.

### 1. Targeting microbial metabolism – Tungstate as a “precision antibiotic”

In inflamed guts, *Enterobacteriaceae* often bloom by exploiting alternative respiration pathways using nitrate or other electron acceptors generated during inflammation. A 2018 study by *Zhu et al.* identified that molybdenum-cofactor-dependent enzymes are critical for *E. coli* and related bacteria to respire anaerobically in an inflamed environment [38]. By administering sodium tungstate, which acts as a competitive inhibitor for molybdenum in bacterial enzymes, they selectively inhibited these pathogens’ respiration. In a mouse colitis model, tungstate treatment sharply reduced the growth of *Enterobacteriaceae* without significantly affecting other commensals. The treated mice showed decreased intestinal inflammation and a microbiota composition shift toward normal. Remarkably, this approach functions almost like a targeted antibiotic in that it zeros in on bacteria utilizing the specific pathway (in this case, those that require the molybdenum-dependent respiratory enzymes during IBD-related dysbiosis). Because most beneficial anaerobes (like *Firmicutes* that ferment fiber) do not rely on these pathways, they are spared. Tungstate is now being optimized for better efficacy and safety, and further studies are examining whether long-term suppression of *Enterobacteriaceae* leads to sustained remission or if bacteria develop compensatory pathways [38, 39]. So far, resistance is less likely because the target is a metabolic requirement rather than a single enzyme. However, potential off-target effects on the host must be monitored, as tungstate can be absorbed and might affect host molybdoenzymes if used chronically.

### 2. Blocking adhesion and colonization – FimH inhibitors

Another pathogenic mechanism in IBD is the ability of certain bacteria to adhere to and invade the gut mucosa. AIEC, often found in Crohn’s ileal lesions, attach to intestinal cells via the type 1 fimbrial adhesin FimH. FimH on bacteria binds to mannose residues on host cells, facilitating colonization of the epithelium and mucosal biofilm formation. This interaction triggers host inflammation and is implicated in post-operative Crohn’s recurrence [40, 41]. A novel small molecule named TAK-018 (also known as EB8018 or “glycomimetic”) was designed to specifically block FimH receptors on AIEC [41]. By occupying the bacterial lectin, it prevents AIEC from sticking to gut mucosa, thereby dislodging them into the lumen where they can be flushed out. Importantly, FimH is largely conserved in *E. coli* and some related coliforms, but blocking it does not kill the bacteria outright; it simply prevents their pathological niche establishment. In a Phase 1b trial in Crohn’s patients with previous surgery, TAK-018 was well tolerated and showed a trend toward reducing AIEC abundance in stools and delaying endoscopic recurrence (though patient numbers were small) [42]. A Phase 2a trial was initiated to evaluate its potential in preventing postoperative Crohn’s disease recurrence, but the study was terminated early due to recruitment challenges, and no efficacy data have been reported [43]. It’s worth noting that FimH is just one example; other virulence factors (like *Clostridioides difficile* toxin receptors, or quorum-sensing signals in *Proteus*) could similarly be targeted by small molecules to disarm pathogens without wiping out commensal microbes [40].

### 3. Bacteriophage (phage) therapy

Bacteriophages (viruses that infect bacteria) are highly specific weapons against bacterial strains. Phage therapy has a long history of use in Eastern Europe to treat infections, and it is now being revisited globally as antibiotic resistance rises [44]. In IBD, phages offer a way to knock down specific bacteria implicated in inflammation. Two bacteria of interest are AIEC and *Klebsiella pneumoniae*, both frequently noted in IBD dysbiosis. A pioneering study by *Federici et al.* demonstrated that an oral cocktail of five lytic phages targeting *Klebsiella* could selectively reduce *Klebsiella* populations in mice, leading to lower gut inflammation [45]. This phage consortium avoided disturbing other gut microbes and did not provoke obvious immune reactions in the host. Crucially, the authors used a combination of phages to prevent the emergence of phage-resistant mutant bacteria, a common challenge in phage therapy. Building on such work, an engineered phage product called EcoActive™ has entered Phase 1/2a trials in inactive Crohn’s disease [46]. EcoActive™ is an orally delivered phage cocktail designed to seek out AIEC in the gut and lyse them. The trial will assess safety and whether AIEC levels and inflammatory markers decrease in treated patients.

However, phage therapy faces unique hurdles. One is the rapid development of phage resistance by bacteria, often via loss or modification of the phage’s receptor on the bacterial surface [47]. This can sometimes make the bacteria less fit or virulent, but it means a single phage is rarely a long-term solution. As noted, using phage cocktails and phage “libraries” that can be rotated are strategies to mitigate resistance. Another issue is delivery and persistence, as phages may be inactivated by stomach acid or cleared quickly; formulations containing buffers or encapsulation help ensure the phages reach the intestine [48, 49]. The gut immune system can also produce anti-phage antibodies if phages translocate or are repeatedly administered, which could potentially neutralize them. This is where synthetic biology and artificial intelligence (AI) come into play. Researchers are exploring engineered phages that evade the immune system or that carry payloads (like biofilm-degrading enzymes) to enhance efficacy [50]. AI can help identify phages from genomic databases that best match a patient’s bacterial strains (a personalized phage selection approach) [51–53]. There are even efforts to program phages with CRISPR-Cas systems to selectively kill bacteria harboring certain antibiotic resistance or virulence genes [54]. In summary, phage therapy for IBD is still in its early days, but it is appealing as a highly targeted antimicrobial that could be used intermittently to prune harmful bacteria while sparing the rest of the microbiome.

### Clinical outlook

#### Current standing and therapeutic potential

Selective pathogen inhibition strategies are likely to play a supportive role in IBD therapy, rather than acting as a cure, given the multifactorial nature of the disease. Their primary potential lies in their ability to target specific disease triggers without causing the collateral damage associated with broad-spectrum antibiotics. Encouragingly, many of these agents (tungstate, receptor blockers, and phages) appear to have favorable safety profiles with minimal systemic exposure.

#### Key challenges and hurdles

A key limitation is that removing specific bacterial strains on their own might not be sufficient to resolve IBD. From a regulatory standpoint, developers will need to provide robust proof that these targeted therapies do not harm the microbiome’s beneficial members or lead to unintended consequences, such as opportunistic infections. Another consideration is patient acceptance, which may need to be addressed through clear education and positive clinical trial outcomes.

#### Future directions and vision

The future of these therapies lies in their use within combination and personalized treatment plans. The vision is a “multi-hit” strategy where these targeted approaches could be used alongside immunosuppressants or microbiome-rebuilding therapies. For instance, a patient might receive biologics to control inflammation, followed by a FimH blocker or phage cocktail to suppress *E. coli*, while concurrently taking a consortium LBP to restore beneficial microbes. This makes personalized microbiome profiling essential, as not all patients harbor the same pathobionts that would be targeted by these specific inhibitors.

In summary, selective microbial inhibition is an emerging precision tool in the microbial therapeutics arsenal, offering a way to “edit out” the harmful actors from the gut ecosystem as part of a broader treatment regimen.

## Microbiota engineering: therapeutic designer bacteria

Instead of adding or removing entire bacteria from the gut, a cutting-edge approach is to genetically modify bacteria to perform therapeutic functions in situ. These engineered bacteria (sometimes called “bacteria as drugs” or next-generation probiotics) are living microbes programmed to deliver beneficial molecules, interfere with pathogenic signals, or otherwise modulate the gut environment in a favorable way [55]. Several pioneering examples in IBD have demonstrated the feasibility of this approach, though translating them to clinical use involves substantial regulatory scrutiny.

### 1. Engineered commensals and probiotics

Early work in this field used food-grade lactic acid bacteria, like *Lactococcus lactis*, as a chassis for delivering anti-inflammatory cytokines [56]. A landmark 2000 study by *Steidler et al.* engineered *L. lactis* to secrete human interleukin-10 (IL-10), a cytokine that suppresses inflammation. In mouse models of colitis, oral administration of these IL-10-producing bacteria led to a significant reduction in inflammation and injury [56]. The approach reached a Phase 1 clinical trial (product known as AG011), which demonstrated safety in Crohn’s disease patients [57]. However, a subsequent Phase 2a trial failed to show efficacy in active Crohn’s, likely because the levels of IL-10 delivered were insufficient (the bacteria did not colonize for long enough or produce sufficient IL-10 in humans) [58]. Nonetheless, this was a milestone: it was the first use of a genetically modified organism (GMO) bacterium in patients to treat disease, proving it can be done safely. Building on that, other cytokine-delivery strains were developed: *L. lactis* has been engineered to secrete IL-27, another regulatory cytokine, which has been shown to attenuate colitis in mice [59]. Similarly, strains secreting soluble tumor necrosis factor (TNF) receptors or single-chain antibodies to neutralize TNF (a key inflammatory cytokine in IBD) have been tested in preclinical models [60]. An *L. lactis* secreting an anti-TNF nanobody was able to reduce inflammation in a chronic colitis mouse model comparable to systemic anti-TNF antibody therapy [60]. These results hint that engineered microorganisms could locally deliver biologic therapy in the gut, possibly reducing the systemic side effects of injectable biologics. Another promising chassis is the well-known probiotic *E. coli* strain Nissle 1917, which is naturally a gut colonizer and has a long safety record [61–63]. Scientists have “upgraded” Nissle to tackle IBD in multiple ways. One approach was endowing Nissle with the ability to produce elafin, an anti-protease that protects tissues from the excess protease activity found in inflamed IBD mucosa [61]. The modified Nissle, when given to mice with colitis, released elafin in the gut, which led to reduced inflammatory damage and a restoration of gut microbial balance. In another approach, Nissle was engineered to secrete a synthetic matrix-binding molecule that forms a protective biofilm coating on the gut lining, thereby preventing harmful bacteria from contacting the epithelium [64].

*Bacteroides ovatus*, a common human commensal, has been engineered to produce transforming growth factor beta-1 (TGF-β1), a cytokine involved in wound healing and immune regulation [65]. Since *B. ovatus* thrives on dietary fiber (xylan), the idea is to administer the engineered *Bacteroides* along with a xylan-rich diet; the bacteria then bloom and deliver TGF-β1 at sites of injury in the colon. Indeed, in mouse experiments, this approach reduced colonic inflammation and promoted mucosal repair [65].

### 2. Synthetic circuitry and sensing

Advanced designs incorporate genetic circuits that make bacteria respond to their environment. For example, an engineered *E. coli* can be equipped with a sensor for inflammation, detecting cues like reactive oxygen species or inflammatory mediators, and then trigger production of a therapeutic peptide only when inflammation is present. An elegant demonstration of this used *E. coli* with a genetic switch that, in high inflammation, causes the bacteria to self-destruct and release anti-inflammatory payload (ensuring the treatment is concentrated during flares and limiting bacteria overgrowth) [66]. There are also “kill switches” embedded in many engineered strains to ensure they do not persist indefinitely in the environment. This is one strategy to address biosafety concerns: engineering strains that are biocontained and cannot establish in nature or transfer their genes.

### Safety and regulatory considerations

Engineered bacteria are classified as GMOs, raising understandable safety and ethical questions. Regulatory agencies will demand rigorous containment measures and proof that the modified microbes will not transfer their new genes to native microbiota (horizontal gene transfer) [67, 68]. So far, the engineered probiotics tested (such as *L. lactis* IL-10) have not exhibited gene transfer or persistence issues; in fact, they typically disappear from stool within days after stopping therapy [57, 69]. To further improve safety, researchers propose “deadman switches”, genetic modules that cause the bacterium to die after a set number of cell divisions, or if antibiotic X is not present [70, 71]. Another approach is using auxotrophic strains (as with *L. lactis* LL-Thy12, which needs thymidine supplementation) so they cannot grow outside a controlled environment [69]. Nevertheless, public perception and ethical oversight are important factors, as releasing GMOs into patients may trigger concerns. However, the field of gene therapy has paved the way to some extent [72]. For IBD, regulators will scrutinize the off-target effects: could engineered bacteria overproduce a molecule and cause local or systemic issues? For example, IL-10 is generally anti-inflammatory, but too much could theoretically impair host defenses. Therefore, precise dosing control is crucial, and many designs use inducible promoters to control how much of the drug is produced [66].

### Clinical outlook

#### Current standing and therapeutic potential

Although still in the experimental stage, engineered bacteria hold great therapeutic potential. Their core advantage lies in being naturally home to the gut, which is an ideal setting for treating IBD. This approach could enable the delivery of combination therapeutics, where a single engineered strain might secrete two or three different beneficial factors simultaneously.

#### Key challenges and hurdles

Before clinical deployment, more human trials are needed to gauge efficacy properly. The setback of the IL-10-secreting bacteria trial was instructive, highlighting that achieving sufficient localized drug delivery is a key challenge to overcome. While newer strains with more potent expression or improved colonization might solve this limitation, it remains a primary hurdle. Success will also depend on continued research, close safety monitoring, and transparent dialogues with both regulators and the public.

#### Future directions and vision

Initially, engineered bacteria will likely supplement, not entirely replace, current treatments. They could be used to manage mild-to-moderate IBD, thereby reducing reliance on systemic immunosuppressants, or be applied during remission maintenance to promote mucosal healing and prevent relapse. The long-term vision is for personalized cocktails of engineered microbes tailored to an individual’s needs: For instance, one strain releasing anti-TNF nanobodies and another producing a mucus-strengthening peptide. These “living therapies” could adapt in real-time to disease fluctuations, a fundamentally different approach from the static dosing of conventional drugs. As our understanding of IBD pathogenesis deepens, new therapeutic targets for these microbes will continue to emerge, and there is also growing interest in using them as vaccines or tolerizing agents [73]. If successful, engineered bacteria could provide highly specific therapy with minimal systemic exposure, essentially achieving immune modulation “from within”.

## Extending the scope: archaea, protists, bacteriophages, and fungi

**Fig. 2:**
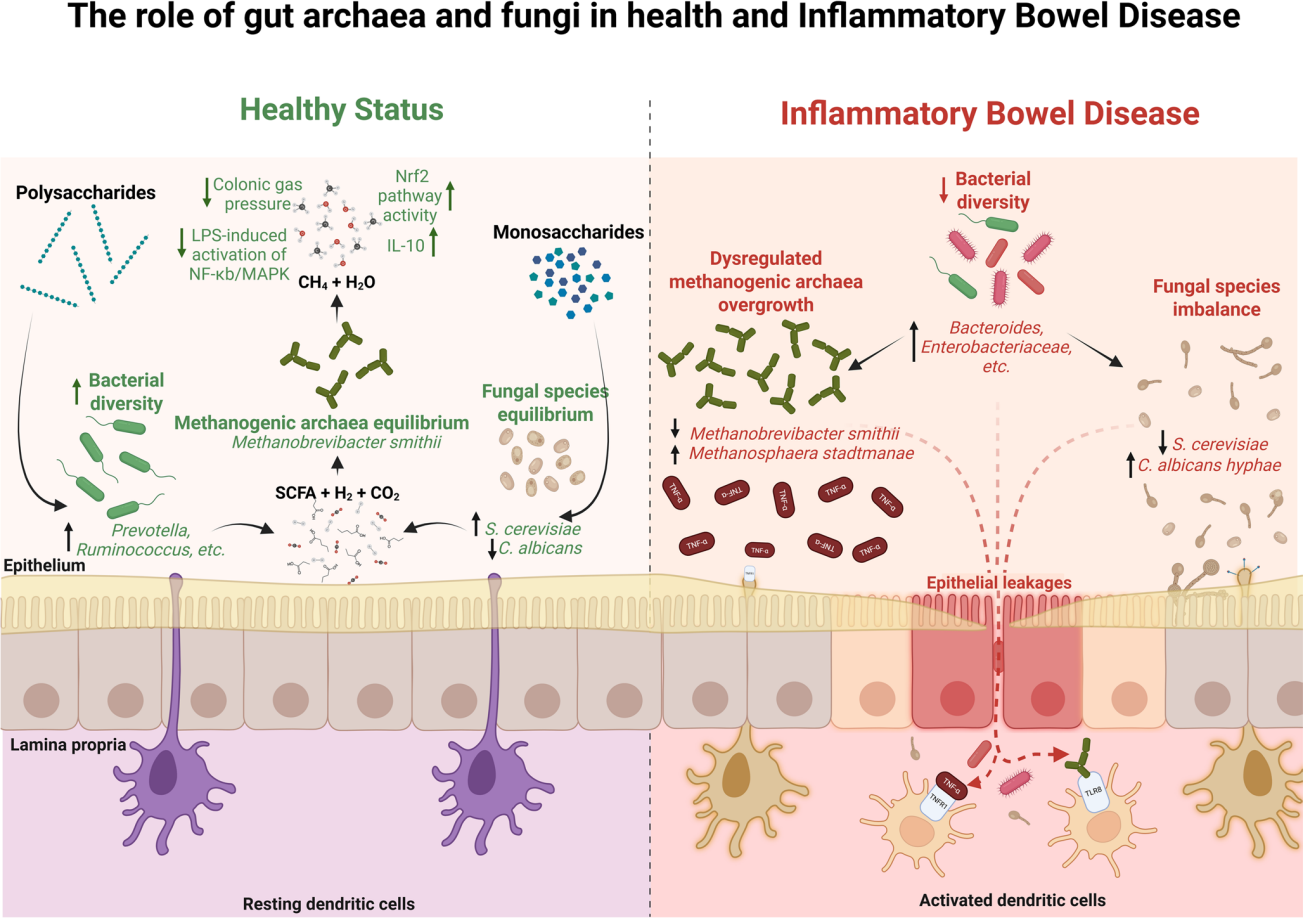
Comparative overview of the dynamics in microbiota composition in the healthy state and in IBD. On the left, a balanced gut ecosystem where methanogenic archaea, a balanced set of fungi, and a diverse array of bacteria coexist in a syntrophic relationship. This balance supports the production of SCFA, hydrogen, and carbon dioxide, which are then converted to methane and water, reducing intraluminal colonic pressure and promoting antioxidant and anti-inflammatory responses. On the right, the disrupted state characteristic of IBD, where bacteria dysbiosis contributes to the overgrowth of methanogenic archaea and an imbalance in fungal species, generating epithelial leakages. This dysbiosis is accompanied by an impaired immune response, with activated dendritic cells intensifying the inflammation and further compromising the epithelial barrier function. Created with BioRender

The human gut microbiome is an ecological network comprising not just bacteria but also domains of life like archaea, diverse protists (single-celled eukaryotes), the virome (especially bacteriophages), and fungi (the mycobiome) [74, 75]. Historically, IBD microbiome research focused heavily on bacteria, largely due to earlier technological limitations in detecting and characterizing non-bacterial organisms [76–78]. However, advances in next-generation sequencing, metagenomics, metatranscriptomics, and improved culture methods have started to illuminate these other domains. It is now clear that archaea, protists, phages, and fungi are not mere bystanders; they interact with bacterial communities and host immunity in important ways [79]. Dysbiosis in IBD extends to these components as well, often in domain-specific patterns, and they might represent both biomarkers of disease and targets for therapy [80]. In this section, we expand on each of these less-explored microbiome members, discussing their known roles in IBD and how they might be leveraged or modulated in future therapeutic strategies (Fig. 2).

### Archaea

Archaea are single-celled microorganisms distinct from bacteria, known for their unique evolutionary lineage and metabolic capabilities [81–83]. In the gut, the most prevalent archaea are methanogens: organisms that produce methane by consuming hydrogen and carbon dioxide. The archetypal human gut archaeon is *Methanobrevibacter smithii*, which can make up a substantial fraction of the anaerobic microbes in some individuals [74]. By scavenging hydrogen (a fermentation byproduct) and producing methane, *M. smithii* and other methanogens perform a “gas sink” role that can improve the efficiency of bacterial fermentation and energy harvest [84, 85]. In a healthy gut, this syntrophic partnership (hydrogen producers and hydrogen consumers) is thought to help maintain a balanced environment. Indeed, lower abundance of *M. smithii* has been observed in active IBD and correlates with more inflammation [86]. The hypothesis is that without sufficient methanogens, hydrogen buildup might impair bacterial fermentation of fibers (leading to less SCFA production) and potentially favor alternative, less beneficial microbial pathways. On the other hand, not all archaea are beneficial. Another gut methanogen, *Methanosphaera stadtmanae*, has been reported at increased levels in IBD patients [87, 88]. Intriguingly, *M. stadtmanae* seems to provoke a strong immune response: laboratory studies showed that *M. stadtmanae* can hyper-activate human dendritic cells, leading to release of pro-inflammatory cytokines like TNFα, IL-6, and IL-1β [88]. *Bang et al.* demonstrated that *M. stadtmanae* stimulated much more robust inflammatory signaling in immune cells than *M. smithii* did [88]. This suggests *M. stadtmanae* might act as a pro-inflammatory driver in the gut if present in high amounts, essentially an archaeal pathobiont. Supporting this, an analysis found *M. stadtmanae* prevalence is significantly higher in both CD and UC patients compared to controls [87].

#### Archaeal dysbiosis in IBD

Large-scale sequencing efforts are starting to map archaeal changes in IBD. A meta-transcriptome analysis that aggregated gut RNA-seq data (the TaMMA framework) found disease-specific differences in archaeal populations [89]. In CD ileum, orders like *Nitrosophaerales*, *Haloferacales*, *Natrialbales*, and *Thermococcales* (some typically found in extreme environments) were unexpectedly abundant. In contrast, UC showed a dominance of methanogenic orders (e.g. *Methanobacteriales* and *Methanomicrobiales*), especially in the colon, with *Methanomicrobiales* being significantly higher in UC colon compared to healthy colons. No significant archaea differences were seen in CD colons in that study, emphasizing differences by location. These findings hint that archaea may not just globally increase or decrease in IBD, but rather their community structure shifts in a context-dependent manner, depending on disease type and gut region [89].

#### Functional impact

Archaea can modulate the gut milieu in ways that affect both host and other microbes. Methanogens like *M. smithii* improving fermentation might correlate with higher butyrate levels (SCFA). Conversely, if *M. stadtmanae* is triggering dendritic cells, it could exacerbate inflammation or break tolerance. Additionally, archaea and bacteria engage in cross-feeding (syntrophy): one produces what the other consumes. Disrupting this partnership might have ripple effects on community metabolism [87, 90]. For instance, some fiber-fermenting bacteria produce hydrogen and acetate that methanogens use; if methanogens are absent, those bacteria might be inhibited by product buildup or might shift to alternative pathways that produce irritating substances. Thus, archaeal dysbiosis could contribute to or sustain the inflammatory state. It remains chicken-and-egg whether archaeal changes cause IBD inflammation or result from it, but at the very least they appear to amplify the dysbiotic state.

#### Clinical outlook

##### Current standing and therapeutic potential

Currently, no IBD therapy directly targets archaea, but several possibilities are emerging. One potential strategy involves supporting beneficial archaea; if a low abundance of *M. smithii* is common in IBD, supplementation could be beneficial. A methanogen-based probiotic might enhance SCFA production or reduce bloating, a concept with precedent in ruminant research [91]. Indirect support could come from diet, as high-fiber diets may favor methanogens by providing more fermentation substrate [92]. Furthermore, it has been noted that individuals with the *Blastocystis protist* (discussed next) have higher archaeal diversity and abundance, hinting at ecological linkages with intervention potential [93].

An alternative is to suppress harmful archaea. If *M. stadtmanae* is proven to be a culprit, targeted approaches might be warranted. Although these ideas are nascent, they could involve archaeal viruses (archaeaphages) [94], designing peptides to block archaeal cell walls [95], or inhibiting archaeal methanogenesis with compounds like statins [96]. Diet may also play a role by limiting the growth of these species by altering the availability of methanol and hydrogen.

##### Key challenges and hurdles

The primary challenge is that this field is still in its infancy. There is a fundamental lack of specific tools, as no archaea-specific drugs are currently available. Furthermore, developing an archaea-based probiotic is difficult, as administering live archaea presents significant technical challenges related to their requirements for anaerobic culture and specific nutrients. More broadly, much more research is needed to clarify the exact role of each archaeon in IBD pathogenesis before therapeutic manipulation can be safely undertaken.

##### Future directions and vision

In the immediate future, archaea are most likely to be useful as biomarkers [97]. For example, combining breath methane tests with stool analysis for *M. stadtmanae* might help identify an IBD subtype with aggressive inflammatory activity, which could influence patient monitoring and stratification in clinical trials. The long-term vision is for future microbiome therapies to consider archaeal support or control as an integral part of achieving true eubiosis, recognizing that these organisms are a key part of gut ecology.

### Protists

Protists are single-celled eukaryotes, some of which reside in the human gut. They include well-known parasites (e.g. *Giardia*,* Entamoeba histolytica*) that cause acute illness, but also commensal or opportunistic protists whose effects are more subtle. The most prevalent intestinal protist in humans is *Blastocystis*, found in 10–20% of people in developed countries and up to ~ 50% in developing regions [98]. *Blastocystis* has many subtypes (at least 9 in humans, labeled ST1–ST9) with considerable genetic diversity [99]. For decades, *Blastocystis* was generally deemed a pathogen, but that view is being re-evaluated as emerging data suggest it may often be a benign commensal or even beneficial under certain conditions [100–102]. Another common protist is *Dientamoeba fragilis*, though its role is less clear [103].

#### Protists in IBD

**Fig. 3:**
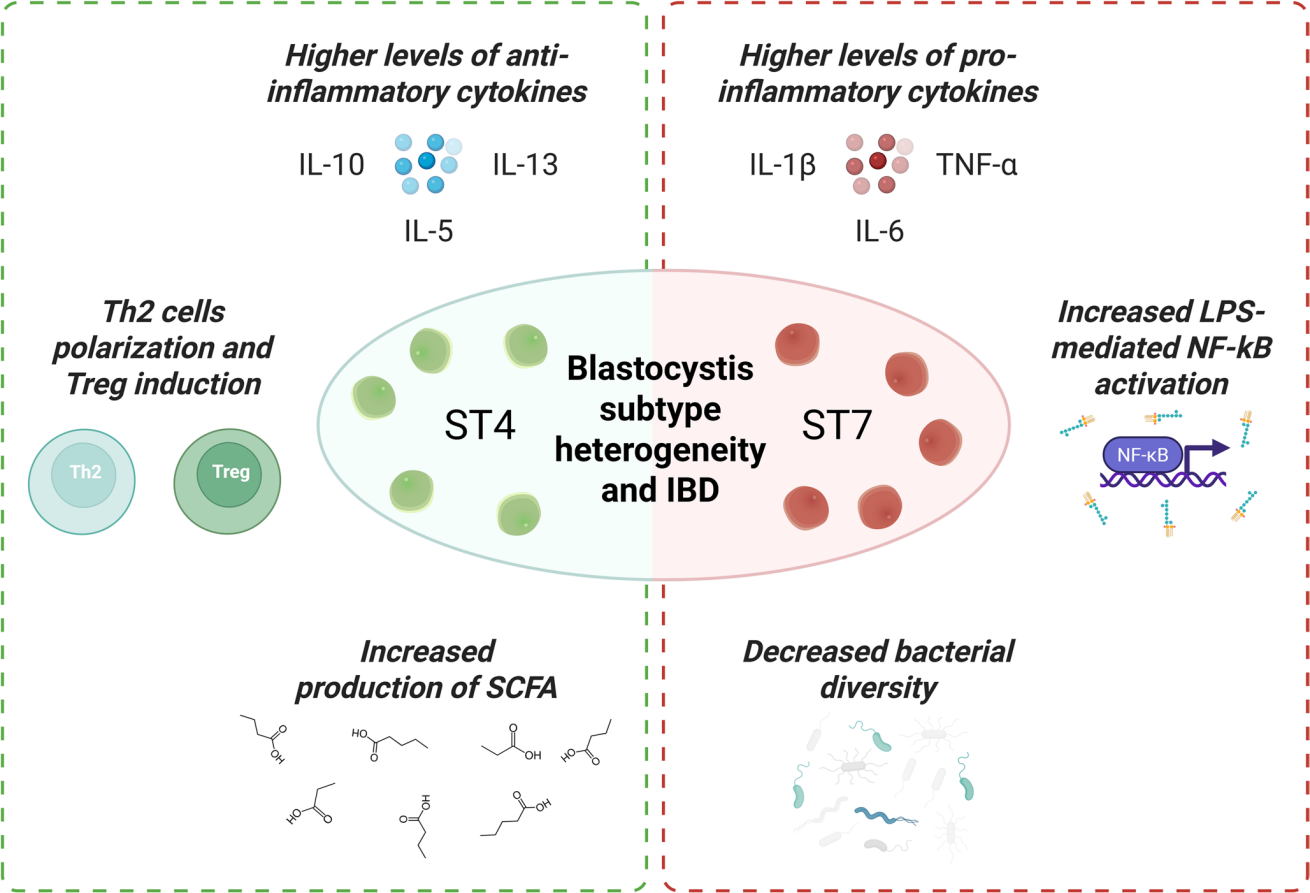
Differential immune effects of Blastocystis subtypes. In the left panel, subtype ST4 colonization promotes a protective environment with increased SCFA-producing bacteria, expansion of colonic Th2 and Treg cells, and higher IL-10, IL-5 and IL-13 levels, leading to suppressed colitis. In the right panel, subtype ST7 infection skews toward a pathogenic environment with enrichment of inflammatory bacteria, activation of Th1/Th17 cells with TNF-α, IL-1β, and IL-6 production, and potentiation of LPS‑mediated NF‑κB activation in monocytes, exacerbating inflammation. These opposing effects underscore the need to distinguish protist subtypes in IBD management. Created with BioRender

*Blastocystis* is less frequently detected in IBD patients compared to healthy controls [104, 105]. Indeed, *Blastocystis*-colonized individuals tend to have higher gut bacterial diversity and a microbiota composition skewed away from the “dysbiosis” pattern [93, 106]. Colonization by *Blastocystis* has been associated with lower abundance of *Bacteroides* and *Enterobacteriaceae* (groups often high in Western dysbiosis and IBD) and higher abundance of *Clostridia* and other beneficial groups [107–111]. However, not all *Blastocystis* are equal. Research has revealed subtype-specific differences (Fig. 3):

- Subtype ST4 (common in Europe) has been linked to anti-inflammatory effects. A series of experiments by *Deng et al.* demonstrated that colonizing mice with *Blastocystis* ST4 actually protected them from chemically induced colitis [112, 113]. ST4-colonized mice showed higher levels of SCFA-producing bacteria and increased proportions of Foxp3^+^ regulatory T cells and IL-10 production in the colon. ST4 seemed to skew the mucosal immune response towards a Th2/Treg profile and away from a pro-inflammatory Th1/Th17 profile. In practical terms, ST4 colonization led to faster recovery from colitis in these models. Fascinatingly, when the gut microbiome from an ST4-colonized mouse was transferred via FMT to another colitic mouse, it conferred protection, implying ST4’s benefit is mediated through modulating the bacterial community and immune milieu [113]. These findings position ST4 as potentially mutualistic: it fosters a microbiome environment and immune state that are anti-inflammatory.
- Subtype ST7, on the other hand, has shown a more pathogenic side. Work by the same group found that ST7 infection in mice exacerbated colitis, with those mice exhibiting more weight loss and gut damage [112]. ST7-colonized mice had an increase in pro-inflammatory IL-17 A and TNF-α producing T cells and a higher abundance of bacteria considered harmful. In vitro studies similarly reported that ST7 isolates can induce human intestinal epithelial cells to produce inflammatory cytokines via activating MAPK pathways (ERK/JNK) [114]. Clinically, ST7 has been linked to diarrheal illness in some reports, whereas ST4 is often found in asymptomatic individuals [102, 115].

For IBD therapy, this suggests that removing protists indiscriminately might not always be beneficial. For example, if a patient’s gut harbors ST4, treating it with antiparasitic drugs could theoretically remove a protective influence and possibly worsen dysbiosis. On the other hand, if a patient has ST7, treating it could remove a pro-inflammatory driver. Currently, there is no routine testing of IBD patients for *Blastocystis* subtype, but this could be an interesting area for personalization. A recent study even posited that colonization with *Blastocystis* ST1 in a mouse model improved metabolic health [116].

#### Clinical outlook

##### Current standing and therapeutic potential

The emerging view of protist heterogeneity in IBD suggests two opposing therapeutic strategies. The first is an intentional inoculation with a benign subtype like *Blastocystis* ST4, which could act as a eukaryotic probiotic to bolster microbiome diversity and promote immune regulation. Conversely, if evidence continues to show that subtypes like ST7 contribute to flares, then targeted eradication of these specific protists might become part of IBD therapy. A third angle involves recognizing the interplay between protists and bacteria; if *Blastocystis* fosters a diverse microbiome, then interventions like consortia or diet that mimic this effect could achieve similar benefits.

##### Key challenges and hurdles

A primary hurdle for any inoculation-based strategy is safety, as a thorough understanding of the risks would be needed before a live eukaryote is administered, requiring careful control and the use of well-characterized strains. Current clinical practices also present a challenge. For instance, the potential failure of some FMTs could be due to the accidental transfer of a pathogenic protist. Meanwhile, current FMT donor screening often excludes individuals positive for *Blastocystis*, a practice that may need re-examination given that some subtypes could be beneficial and that an inadvertent transfer showed no inferior clinical outcomes [117].

##### Future directions and vision

As we strive for precision medicine, the ultimate vision is for personalized protist profiling to inform therapeutic strategies. Understanding the immune system’s response to different protists could also unveil novel pathways to exploit for treatment. The focus must also expand beyond *Blastocystis* to other commensals like *Entamoeba coli* and *Chilomastix mesnili*, which are often found in contexts of high microbial diversity and gut health [118]. Ultimately, encouraging a tolerant co-existence with a variety of commensal protists may be a key component of restoring a balanced and healthy gut ecosystem in IBD patients.

### Virome

The term “virome” encompasses all viruses in the gut, the majority of which are bacteriophages that infect bacteria. The healthy human gut has a rich phage community, often specific to each individual, which co-evolves with the bacterial microbiome [119]. Phages can exist in two states: lytic (destroying their bacterial host cell to release new phages) or lysogenic (integrating into the bacterial genome and replicating quietly as the bacterium divides). In a stable gut, many phages are believed to be temperate (lysogenic), maintaining a sort of detente with their hosts. Studies have found that in healthy conditions, the virome is dominated by certain double-stranded DNA phage families (like *Caudoviricetes* order, encompassing classical tailed phages) and smaller ssDNA phages (*Microviridae*) [120].

#### Virome in IBD

In IBD, the virome appears dysregulated alongside the bacterial dysbiosis. A consistent observation is an increased phage richness and altered composition in IBD patients’ guts [121, 122]. Specifically, several studies found that *Caudovirales* (tailed phages) are overrepresented in IBD, while *Microviridae* (like *CrAssphage* and others that typically infect *Bacteroidetes*) are relatively depleted [123]. Moreover, phages in IBD tend to shift towards the lysogenic-to-lytic ratio: more temperate phages might be induced into lytic cycles due to the stressed environment [124, 125], or conversely, the environment might favor lysogens of certain species [123, 126, 127]. Another intriguing finding is that expanded phage activity could directly contribute to bacterial depletion: for instance, if beneficial bacteria are being lysed by an expanding population of their phages, that could exacerbate loss of diversity in IBD. An example: *Faecalibacterium prausnitzii*, a beneficial anti-inflammatory bacterium, is often reduced in Crohn’s patients. It has been speculated that Crohn’s patients may harbor specific *Faecalibacterium*-phages that bloom and kill off these bacteria [128]. In fact, one study identified certain Caudovirales associated with IFN-γ production that were enriched in IBD, hinting that phages might influence immune responses either directly or via shaping bacteria [129]. The mechanistic link could be that when phages lyse bacteria, they release microbial DNA and antigens that stimulate innate immunity. Inflammation can cause bacteria to release phages (through SOS response induction of prophages), so flares might trigger bursts of phage activity [123, 130]. As a result, those phages then alter bacterial populations further, perhaps locking in a dysbiotic state [126, 127]. Researchers have coined the term “bacteriophage translocation” for findings of phage DNA in host blood/tissues of IBD patients, suggesting that increased intestinal permeability might let phages cross the barrier and even influence systemic immunity [129, 131].

#### Clinical outlook

##### Current standing and therapeutic potential

The therapeutic potential of the virome extends along two main avenues. The first is the direct use of phages as therapy to eliminate pathogenic bacteria, as previously discussed. The second, more nuanced approach, involves modulating the native phage population itself. This could include strategies like inhibiting the induction of temperate phages known to lyse beneficial bacteria or using phage-derived enzymes like endolysins for precise bacterial killing without administering whole viruses [132, 133]. Other forward-looking concepts include using phages as delivery vehicles for vaccines [134], or pushing the phage community toward lytic phages that self-limit once their host is eliminated.

##### Key challenges and hurdles

The primary challenges for phage-based therapies are rooted in their complex and dynamic nature. Regulatory and safety issues are significant because phages can evolve rapidly and mediate horizontal gene transfer between bacteria [135]. This carries the risk of inadvertently enhancing pathogen virulence by transferring toxin or antibiotic resistance genes, a particular concern in IBD where viromes show a higher potential for such gene transfers [127]. Furthermore, the native virome presents a practical hurdle for other microbiome therapies, as pre-existing phages in a patient’s gut could potentially attack and neutralize an administered bacterial consortium.

##### Future directions and vision

An immediate and practical step is to monitor the virome as a standard part of microbiome assessments [136]. In the near future, the virome holds great promise as a diagnostic tool. Several studies have identified distinct virome signatures that can differentiate between Crohn’s disease, ulcerative colitis, and healthy states, which could be used to identify IBD types or predict flare risk in a domain known as “virobiota profiling“ [126, 127, 136–138]. Understanding the virome will also be crucial for optimizing other treatments; for example, the success of FMT may partly depend on the successful transfer of a healthy phage community from the donor [139, 140].

In summary, the microbial network in IBD includes viruses as key players. They often act in a predator-prey dynamic with bacteria, and a stable equilibrium is part of health. IBD seems to alter that equilibrium, leading to phage expansions that possibly perpetuate dysbiosis.

### Fungi

The gut mycobiome is less abundant (by orders of magnitude) than the bacteriome, but fungi like *Candida*, *Saccharomyces*, and *Malassezia* are regularly present. They form part of the normal commensal flora, though most are transient from diet (yeasts in food) or environment [141]. In a healthy gut, fungal populations are kept in check by immune mechanisms (e.g. Dectin-1 receptor on myeloid cells recognizing fungal cell wall components) and by bacterial competition [142, 143].

#### Mycobiome changes in IBD

IBD patients often exhibit quantitative and network alterations in gut fungi. A notable finding is an increased relative abundance of *Candida* species (especially *Candida albicans*) in both pediatric and adult IBD [144–147]. One study found that during active Crohn’s, *Candida* was more frequently isolated from stools, and anti-*Saccharomyces cerevisiae* antibodies (ASCAs) are elevated in many Crohn’s patients [148]. This suggests an ongoing immune exposure to fungal antigens in IBD [149]. Fungal diversity reports have been mixed: some studies say diversity increases in IBD, others say specific lineages like *Basidiomycota* (e.g., *Malassezia* from skin that might colonize gut) go up while *Ascomycota* shift [150, 151]. Despite inconsistencies, it’s agreed that fungal–bacterial network interactions are perturbed [145–147, 152]. A study by *Sokol et al.* showed that in healthy individuals, fungal and bacterial communities form an interconnected network, whereas in IBD this network is altered with *Candida* dominating and a loss of normal correlations [145].

#### Host-fungal immune interactions

Host genetics highlight fungi’s relevance: Polymorphisms in CARD9 (an immune adaptor for fungal sensing) are risk factors for IBD [153, 154]. CARD9-deficient mice cannot clear *Candida* well and develop worse colitis, which is ameliorated by anti-fungal treatment, linking fungal persistence to inflammation [155]. Similarly, Dectin-1 (CLEC7A) is a receptor for fungal beta-glucans; a Dectin-1 polymorphism has been associated with severe UC. Mice lacking Dectin-1 have worse colitis marked by higher *Candida* and *Aspergillus* burdens [142]. These data imply an IBD scenario where a defective anti-fungal immune response leads to fungal overgrowth that fuels inflammation. Indeed, IBD patients often have antibodies against *Saccharomyces cerevisiae* (ASCA), possibly because of abnormal immune exposure to gut fungi [156]. Those with CARD9 mutations might particularly benefit from anti-fungal strategies, as their immune system fails to normally handle fungi.

#### Causality evidence

A striking experiment showed that colonizing germ-free mice with a specific *Candida albicans* strain induced intestinal inflammation, but only if the strain could transition to its invasive hyphal form [157]. A hyphal-deficient *Candida* mutant did not cause the same inflammation. This suggests fungal morphology and virulence factors are triggers for the immune system. In humans, Crohn’s lesions have been found to contain *Candida* and *Malassezia*, raising the possibility that they directly contribute to mucosal damage or immune activation [158]. *Malassezia restricta*, normally a skin fungus, was found enriched on the intestinal mucosa of Crohn’s patients (especially those on biologics), and it induced strong cytokine responses via CARD9 pathways [151].

#### Clinical outlook

##### Current standing and therapeutic potential

Several strategies are currently being explored to modulate the gut mycobiome. Antifungal drugs like fluconazole have shown some benefit in small trials, especially in patients with high *Candida* levels [159, 160]. The use of probiotic yeasts is another promising avenue; *Saccharomyces boulardii*, for instance, has demonstrated efficacy in preventing *C. diff* recurrence and may help maintain remission in UC, potentially by outcompeting *Candida* and modulating immune responses [161, 162]. Finally, dietary modulation, such as reducing simple sugars, is a strategy some patients use to limit yeast growth, and it could theoretically complement other therapies [163].

##### Key challenges and hurdles

A significant risk with broad-spectrum antifungals is that they could disrupt beneficial fungi or select for resistant strains. While adjunct antifungal therapy shows potential for individuals with specific risk factors like a CARD9 allele, its efficacy still needs to be validated in larger clinical trials [164]. Similarly, the benefit of low-carb or yeast-free diets currently lacks formal evidence. An additional challenge is that broad-spectrum antibiotic use in IBD can inadvertently spark fungal blooms, meaning that careful antibiotic stewardship is a crucial part of managing the mycobiome.

##### Future directions and vision

The future of mycobiome therapy will likely involve more precise and personalized approaches. This includes speculative but promising ideas like therapeutic vaccines targeting *Candida* virulence factors to boost mucosal immunity in genetically susceptible individuals. Future therapies may also involve precise targeting of *Candida albicans* with monoclonal antibodies or even engineered bacteria designed to secrete fungicides in the gut. It will also be critical to consider cross-domain effects in all microbiome-based treatments. For example, FMT donor selection might benefit from fungal screening, and introducing a bacterial consortium could help re-establish a healthy bacteria-fungi equilibrium, highlighting the need for a holistic view of the gut ecosystem.

## Conclusion

IBD management is poised at an inflection point as we broaden our focus from solely tempering the host immune response to actively remodeling the intestinal microecosystem. The research reviewed herein highlights that the gut microbiome, encompassing bacteria, archaea, protists, viruses, and fungi, is not merely an innocent bystander in IBD but often an instigator and perpetuator of mucosal inflammation. Emerging microbiome-directed therapies offer new hope by aiming to restore a healthy symbiosis in the gut, addressing a dimension of IBD pathogenesis away from the traditional drugs’ scope. From the promising results of rationally designed bacterial consortia in preclinical models and early trials to the advent of targeted phage cocktails and genetically engineered probiotics, we are witnessing the development of a rich therapeutic toolkit that can complement and, in some cases, might even replace standard immunotherapies in the future. A unifying theme across these therapies is the move towards precision medicine. No longer would IBD be managed with a one-size-fits-all approach; instead, treatments will be tailored to an individual’s unique microbial and immunological makeup. This precision will be enabled by advances in diagnostics, routine profiling of the gut microbiome (perhaps even at point-of-care), and host genetic/immunologic markers to guide therapy choices.

Our review also underscores the importance of an integrated, multi-domain perspective. We have refined our understanding that what defines a “healthy” vs. “IBD” microbiome is not just the presence or absence of one microbial group, but the network of interactions among all members of the microbiota and the host. Beneficial taxa can turn harmful under certain conditions and vice versa. Therefore, therapeutic interventions must be context-aware. The concept of a “healthy ecosystem” may serve as an overarching framework: therapies should strive to rebuild an ecosystem characterized by diversity, stability, and functional redundancy, wherein no single pathogenic species can dominate and where host-friendly functions (like butyrate production, mucus integrity, and immune tolerance) are robustly provided by the microbial community. Achieving this may require combining therapies, and we anticipate that the next generation of clinical trials will explore such combinations, bringing us closer to that ecosystem restoration goal.

Of course, there are significant challenges ahead. The regulatory pathways for live microbiome products must be navigated and refined to ensure safety without stifling innovation. Long-term monitoring will be necessary to prevent introduced microbes or phages from evolving in unintended ways or disturbing the balance in the long term. The specter of horizontal gene transfer, especially with GMOs, necessitates robust biocontainment strategies. Ultimately, the vision for the future is a unified, bench-to-bedside paradigm where insights into host-microbe interactions directly inform personalized treatment plans. The convergence of clinical medicine, microbiology, and immunology will usher in a new era in which IBD, a long-standing challenge, can be met with multifaceted and nuanced therapies that offer patients not just temporary relief, but the prospect of long-term remission and improved quality of life.

## Data Availability

Data sharing is not applicable to this article as no new data were created or analyzed in this study.
